# What happens when public primary care is ill-prepared to respond to non-communicable diseases: a mixed-method study of diabetes and hypertension care in urban Nepal

**DOI:** 10.7189/jogh.16.04218

**Published:** 2026-07-10

**Authors:** Deepak Joshi, Helen Elsey, Grishu Shrestha, Raju Neupane, Parash Mani Sapkota, Bikram Adhikari, Sampurna Kakchapati, Santosh Giri, Abriti Arjyal, Shreeman Sharma, Sujan Poudel, Sushil Chandra Baral

**Affiliations:** 1 HERD International, Lalitpur, Nepal; 2 Hull and York Medical School, University of York, Heslington, UK

## Abstract

**Background:**

With rapid urbanisation in low- and middle-income countries, public primary care is struggling to respond to the growing prevalence of hypertension and diabetes, while the private sector has grown to meet demand. We aimed to understand current use and readiness of public and private primary care diabetes and hypertension services, and how readiness impacts perceptions and practices of urban communities.

**Methods:**

We conducted a convergent mixed-methods study in Nepal, including mapping of diabetes and hypertension services in 134 public and private facilities; participatory social mapping, transect walks combined with six focus groups and nine interviews with marginalised urban communities and 15 city and health system actors to understand perceptions and service use. We used descriptive statistics to assess service readiness and thematic analysis of our qualitative and participatory data.

**Results:**

Of the 660 facilities mapped, 134 facilities provided diabetes and hypertension services, and 63% were private. Median service readiness scores were similarly low for hypertension and diabetes across public and private primary care. Trained human resources and the availability of guidelines scored the lowest within this overall score. Qualitative findings highlighted a lack of willingness to take long-term medication, high use of pharmacies, and acceptance of hypertension and diabetes as part of ageing. Attempts to improve behaviours were undermined by the living and working conditions facing marginalised urban communities.

**Conclusions:**

While both public and private primary care services are diagnosing and managing hypertension and diabetes, few have appropriate training or guidelines. This undermines patient trust, with many self-managing through pharmacies and non-allopathic medicines. Limited population and routine health data, coupled with a lack of data about the urban poor, weaken the ability of city and health system actors to plan health services, particularly targeting marginalised city residents.

Rapid urbanisation is a key determinant of health, particularly in relation to non-communicable diseases (NCDs) [1]. The urban environment in low- and middle-income countries (LMICs) is characterised by a dense and growing population, and for many in low-income households, this means living in slum housing or informal settlements as defined by the United Nations-Habitat as overcrowded areas, with households that lack tenure, adequate water, sanitation, and access to essential services [2]. The combination of poor living and working conditions leads to continued exposure to communicable diseases as well as the rising prevalence of NCDs [3]. While originally seen as diseases of the wealthy, there is increasing evidence from LMICs that the social gradient has tilted. Meta-analysis of hypertension and diabetes among slum communities found increasing prevalence across South Asia, with slum residents more likely than their rural counterparts to be hypertensive [4]. Nepal exemplifies rapid urbanisation and growth in NCDs, with a recent review estimating the pooled prevalence of hypertension in slums at 28% (95% confidence interval = 24.72–31.43) [4]. Similar findings are emerging from more recent studies highlighting higher hypertension prevalence among slum residents in Nigeria [5] and Bangladesh [6].

Slum areas – as opposed to slum households – may face environmental risk factors in addition to individual factors due to neighbourhood effects, including exposure to pollutants, poor environments, and stress, caused by the fear of eviction and crime levels [7]. The living and working environment of urban poor and slum households is driving changes in diet, with easily available processed and unhealthy foods coupled with challenges in accessing and cooking fresh vegetables and fruit across LMICs [8] and in Nepal [9]. This is coupled with increasing sedentary lifestyles [10], often with marked gender differences [11], high levels of tobacco and alcohol use, particularly among male slum dwellers and urban poor [12,13]. In Nepal, where analysis of the urban data within the 2019 STEPS survey indicates that while poor, middle, and rich wealth categories have similarly insufficient fruit and vegetable consumption (85–87%) and insufficient physical activity (16–17%), levels of smoking are significantly higher among the urban poor (20%) compared to middle (16%) and rich (15%) urbanites (*P* = 0.003) [14].

With growing urban populations, changing exposures, and rising prevalence of NCDs [15], public primary care in Nepal is struggling to respond, as it was designed to serve smaller populations and has traditionally focused on infectious diseases, maternal, and child health [16]. This means that urban residents, as in many LMICs, frequently rely on the private sector for care [16,17]. This takes the form of private clinics and pharmacies, which have sprung up to fill the gaps left by the public system [17]. However, the private sector across South Asia is largely unregulated and heterogeneous, with limited available data to assess performance and reach [16].

The public urban health system in Nepal includes urban health centres, health posts, and primary health care centres, and these are managed by local governments under Nepal’s federal system. Challenges with equitable access have been noted, with a pro-rich distribution of general health care utilisation among both public and private providers at all levels in urban areas [18]. National surveys highlight gaps in treatment and diagnosis by wealth quintile, showing significantly lower levels of awareness, treatment, and control of hypertension among the lowest wealth quintiles compared to the higher wealth quintiles [19]. The limited free and accessible primary care system in urban areas increases the likelihood of remaining undiagnosed and untreated for common NCDs, such as diabetes or hypertension [20,21]. For those diagnosed, their access to prevention and treatment, and subsequent compliance with treatment services, is inhibited due to lack of access to services and affordability [22-24]. This compounds the impacts that NCDs, particularly when poorly managed, have on economic productivity and along with associated costs for treatment services, NCDs can drive poor people further into poverty [1,25].

We conducted this study as part of a broader research programme aimed at strengthening urban health systems by integrating private health providers with public primary care to improve diabetes and hypertension prevention and care for the urban poor. We utilised a mixed-methods study design to understand primary care provision of diabetes and hypertension services across public and private providers, and the experiences and perceptions of community members in accessing care, preventing and managing these two conditions. Additionally, we aimed to explore the challenges facing city and health system actors in planning to deliver hypertension and diabetes prevention and care to urban poor communities.

## METHODS

### Study design

We used a convergent mixed-methods design integrating quantitative and qualitative results at the end of data collection [26]. We conducted a census mapping of all public and private providers in Pokhara, followed by a quantitative assessment of readiness to deliver diabetes and hypertension services (from February to May 2022). We also conducted a qualitative and participatory study with urban poor communities, healthcare providers, and city decision-makers.

### Study setting

Pokhara is the largest metropolitan city in western Nepal’s Gandaki province and the fastest growing in the country [27,28]. The city’s thriving tourism industry, providing both skilled and unskilled employment, has fuelled internal migration [29,30]. An estimated 40% of Nepal’s urban population resides in informal settlements or slums [31]. However, slums are not the only location of poverty, particularly in the context of Pokhara, where poor, slum households are distributed in many areas of the city. These settlements and clusters of slum housing are crowded, lack basic water and sanitation facilities, have narrow walkways unsuitable for vehicles, and are situated in areas susceptible to river flash floods and adjacent to waste dumping sites.

### Provider mapping and health facility assessment

We mapped all public and private health facilities within the Pokhara Metropolitan City (PMC), using Open Street Map and stakeholder consultation to prepare the list of health facilities. We identified facilities providing diagnosis and/or management services for diabetes and/or hypertension in an outpatient capacity and included them in the assessment sample.

To assess readiness for diabetes and hypertension care, we used the diabetes and cardiovascular disease components of WHO’s Service Availability and Readiness Assessment (SARA) [32]. Using the three items relating to hypertension and the four items relating to diabetes (Tables S1–2 in the **Online Supplementary Document**), we generated readiness scores following approaches used in previous studies [33,34], with a maximum readiness score of 100. These items were selected to align with the GoN’s NCD protocol [35], ensuring a context-appropriate interpretation of ‘readiness’.

### Qualitative and participatory method

We aimed to understand the perspective and experience of city and health system actors and urban poor communities in relation to hypertension and diabetes care. Given the limited data available on the location and characteristics of urban poor populations, local government officials directed us to Wards perceived as having a high proportion of informal settlements and slum housing. We then conducted participatory social mapping and transect walks [36] to identify urban poor neighbourhoods within these wards (**Box 1**, **Box 2**). Through detailed discussions with community members, we identified characteristics of marginalised individuals and households at risk of diabetes and/or hypertension (due to their age of >40 years). Based on these characteristics, we purposively sampled participants for in-depth interviews (IDIs) and focus group discussions (FGDs) (Table S3 in the **Online Supplementary Document**).

Box 1 **Social mapping**

Initially, we held mapping workshops with key informants from civil society, including representatives of slum communities and local government stakeholders with at least five years of experience working in the city, to agree on the characteristics of urban poor settlements and households. These key informants identified predominant occupations and locations (slum areas) as particularly important, and based on this classification, we identified two wards within PMC where poor households/population reside.

Within these identified wards, we conducted social mapping exercises with a wide range of local stakeholders, including female community health volunteers (FCHVs), ward chairpersons, ward secretaries, sub-ward representatives, and health facility managers, as well as community-based organisations. Following discussions in each area, community members agreed on some common key characteristics to identify poor households: whether a household can fulfil their basic needs, housing condition, any challenges in paying for healthcare services during illness, and the source of income/occupation of household members. Households believed to meet these characteristics were then marked on a printed map of the area (based on OpenStreetMap output). We also asked participants to colour-code female-headed households, elderly individuals, those with disabilities and those >40 years old. Along with urban poor households, healthcare-seeking sites, such as public health facilities, but also pharmacies, both registered and non-registered, were added to the map.

There were 5–10 sub-wards reported from each mapping exercise. Based on stakeholder suggestions, we identified one sub-ward from two different wards for further participatory and qualitative work, including transect walks, FGDs, and IDIs.

Box 2 **Transect walk**

The transect walks aimed to build a more detailed understanding of the factors influencing health, particularly lifestyle behaviours and management of diabetes and hypertension, as well as healthcare-seeking behaviour for these conditions. Within these sub-ward clusters (Tole), through snowball sampling with community leaders in the sub-wards, we identified the community members who had lived and/or worked in that community for at least one year. Together with these individuals, we conducted a transect walk around the identified sub-ward area. Transect walks took up to one hour, and the participants discussed living and working conditions of residents, lifestyle behaviours related to diabetes and hypertension and where people seek healthcare. As many residents worked long hours during the day, we also conducted one transect walk in the evening when the neighbourhood was full of activity, including many local bars serving the daily wage labourers after work. After the walk, two researchers (RN and DJ) discussed with the key informants and prepared a list of the household locations. While preparing the list, we considered age and gender as key criteria for FGDs, whereas for IDIs, we additionally considered their disease condition, either hypertension or diabetes. While preparing the list, we attempted all the prompts so none of the eligible households would be missed. From the list, we purposively selected the participants and contacted them further to obtain their consent to participate in the study.

#### IDIs

The interviews focused on the participants’ understanding of diabetes and hypertension, their experience and perceptions of seeking care for diagnosis, advice and treatment using an interview guide, but encouraged participants to talk in-depth on aspects important to them.

#### Key informant interviews (KII)

We conducted 15 KIIs with city and health system actors to understand health system challenges in addressing NCDs in the city using an interview guide. These included seven officials responsible for health planning and policy development within the PMC office and eight healthcare providers delivering diabetes and hypertension care services (Table S5 in the **Online Supplementary Document**).

#### FGDs

Within six FGDs with community members, we used a participatory ranking exercise [36] to understand healthcare-seeking behaviour for NCDs, particularly diabetes and hypertension. We provided the participants with a one-page matrix and asked them to score types of healthcare facilities (using 20 counters) based on distance, affordability of healthcare services, and acceptability of services. Each group reached a consensus, then scored facility types against each criterion. They rated each health facility on a five-point scale, where five (counters) reflects proximity and zero reflects remoteness. We repeated the process for affordability, acceptability, information, and total scores (**Table 3**). The primary purpose of the ranking exercise was to facilitate discussion among the group, rather than the final scores.

**Table 3:** Mean rating score on access to healthcare services across all FGDs

	Distance	Affordability	Acceptability	Information	Total
**Pharmacies**	5	4	4	3	16
**Traditional healers**	5	4	2	2	13
**Private hospitals/clinics**	3	2	4	2	11
**Primary healthcare centres**	4	4	2	2	12
**Public hospitals**	1	3	2	2	8

#### Data collection

Researchers of the same gender as the participants conducted qualitative interviews. A senior researcher (DJ) conducted all KIIs and FGDs with support from two additional researchers (SS and ST), following the study methods and guidelines. All researchers were university graduates in health science with training in qualitative methods. We conducted the data collection in Nepali. We recorded IDIs, KIIs, and FGDs using an audio recorder, keeping notes of the process and emerging issues. Given the challenges of audio-recording in noisy urban streets, we made detailed notes during and after the transect walks and social mapping process.

### Data analysis

#### Qualitative data analysis

Researchers transcribed the interviews within a week of data collection and used notes and audio recordings to check accuracy. We presented the initial findings to the communities for validation, and participants reported agreement with the findings. Four researchers (DJ, RRN, SS, and ST) conducted the preliminary coding process to develop a coding framework using transcripts from KIIs, IDIs, and FGDs. The team manually coded all transcripts using thematic network analysis to generate a network of ‘global’, ‘organising’, and ‘basic’ themes [37]. This was achieved through a series of iterative discussions among the team to identify any duplication within the basic or organising themes. We then explored how these themes related to the field notes from the social mapping and transect walk. We carried out this process until we agreed that the resulting network of global themes, organising themes, and basic themes was distinct. We followed the Standard for Reporting Qualitative Research checklist [38].

#### Quantitative data analysis

We performed descriptive statistical analysis and presented the results of the health facilities assessment in tables and charts. We used frequencies and percentages for categorical variables and means (x̄) standard deviations (SD), and medians (MDs) (interquartile ranges (IQRs)) for continuous variables. In the assessment, we included the readiness of the health facilities to deliver outpatient management of diabetes and hypertension services as the outcome variables. We used the three hypertension and four diabetes items from the SARA tool: staff training and guidelines, equipment, and medicines, with the additional domain on diagnostics for diabetes (Tables S1 and S2 in the **Online Supplementary Document**) to generate the readiness scores [32]. We marked the results for services not specified within the GoN’s protocol for the specific type of service provider as not applicable [35]. We calculated a composite readiness score for each service by giving equal weight to each of the domains and each of the indicators within the domains. This approach is described in the SARA manual [32] and has been used in previous [33,34]. As the GoN’s NCD national protocol presents these components as essential without any prioritisation, we considered equal weighting of individual items to be appropriate within the composite readiness scores.

#### Convergent mixed method analysis

Following the separate quantitative and qualitative analyses, we organised the global themes from the qualitative analysis to allow comparison with the quantitative results, where each row constituted a theme and each column a data source. The final global themes were: health literacy, screening and diagnosis; management of hypertension and diabetes; accessing care; preventative behaviour; and population and planning. Some data sources contributed more to each theme than others; where information for a theme was available across the different data sources, we assessed whether findings were confirmed or disconfirmed across data sources. We presented the combined findings with integrated data from multiple data sources (mapping, facility assessment, qualitative, and participatory) to provide a comprehensive understanding of each global theme (Table S8 in the **Online Supplementary Document**).

## RESULTS

Through the mapping process, we identified 660 health providers in PMC and categorised these into 21 types of institutions based on their services. Of all health institutions in PMC, most were private/non-governmental-operated facilities (92%), of which most were pharmacies (53.6%), while only 8% were public facilities.

Following screening, we found 134 allopathic health facilities to be providing a range of hypertension and diabetes diagnosis and management services (Tables S6–7 in the **Online Supplementary Document**). Of these, the majority were private providers (63%), including private clinics and private hospitals. In addition, we identified 173 pharmacies providing hypertension and diabetes services beyond drug dispensing [39].

### Knowledge, screening, and diagnosis

Our qualitative findings supported and further elucidated our quantitative results. The facility assessment showed that while services, particularly private clinics, had diagnostic equipment available, few were trained or had relevant guidelines to support them to advise or diagnose diabetes or hypertension (**Table 1**, **Table 2**). For public primary care, diagnosis of diabetes was a particular challenge, with only 64% of public primary care services able to assess blood glucose, few staff trained (4.4%), and limited availability of guidelines (4.4%). While more private primary care clinics (91.5%) were able to assess blood glucose, staff training (3.4%) was limited, and none had guidelines available (**Table 1**). For hypertension, public and private primary care had better availability of equipment, with all clinics having a blood pressure gauge, although limited again by a lack of staff training and guideline availability (**Table 2**). Hospitals, both public and private, were better equipped for both hypertension and diabetes diagnosis, but they still lacked trained human resources, particularly within the private sector and for diabetes.

**Table 1:** Diabetes readiness by health facility type*

	Private clinic (n = 59)	Public primary healthcare facilities (n = 45)	Private hospital/nursing home (n = 26)	Public hospital (n = 4)	Overall (n = 134)
**Diagnostic services, x̄ (SD)**	72.9 (25.1)	64.4 (48.4)	94.9 (12.3)	91.7 (16.7)	74.9 (34.8)
Blood glucose	54 (91.5)	29 (64.4)	26 (100.0)	4 (100.0)	113 (84.3)
Urine protein†	41 (69.5)	NA	24 (92.3)	4 (100.0)	69 (77.5)
Urine ketone†	40 (67.8)	NA	24 (92.3)	3 (75.0)	67 (75.2)
**Staff and guidelines overall, x̄ (SD)**	1.7 (9.1)	4.4 (14.4)	1.9 (9.8)	25.0 (28.9)	3.4 (12.6),
Trained human resource	2 (3.4)	2 (4.4)	0 (0.0)	1 (25.0)	5 (3.7)
Government of Nepal PEN guidelines	0 (0.0)	2 (4.4)	1 (3.8)	1 (25.0)	4 (3.0)
**Equipment, x̄ (SD)**	86.4 (17.6)	99.3 (5.0),	100.0 (0.0)	100 (0.0)	93.8 (13.7)
BP set	59 (100.0)	45 (100.0)	26 (100.0)	4 (100.0)	134 (100.0)
Weight measuring equipment	58 (98.3)	45 (100.0)	26 (100.0)	4 (100.0)	133 (99.3)
Height measuring equipment	36 (61.0)	44 (97.8)	26 (100.0)	4 (100.0)	110 (82.1)
**Essential medicines, x̄ (SD)**	72.9 (25.1)	55.6 (15.9)	94.9 (12.3)	91.7 (16.7)	71.9 (24.5)
Metformin	56 (94.9)	45 (100)	26 (100)	4 (100)	131 (97.8)
Glimepiride†	50 (84.7)	NA	25 (96.2)	4 (100)	79 (88.8)
Dextrose	23 (39.0)	5 (11.1)	23 (88.5)	3 (75.0)	54 (40.3)
**Diabetes service readiness, x̄ (SD)**	58.5 (14.7)	55.9 (13.7)	72.9 (6.8)	77.1 (13.8)	61.0 (14.7)

BP – blood pressure, NA – not applicable, SD – standard deviation, x̄ – mean

*Values are presented as numbers (percentages) unless specified otherwise.

†Denominator is 89 facilities instead of 134 since urine protein and urine ketone (diagnostics) and glimepiride (medicines) are not mandated for public PHC in Nepal.

**Table 2:** Hypertension readiness by health facility type*

	Private clinic (n = 59)	Public primary healthcare facilities (n = 45)	Private hospital/nursing home (n = 26)	Public hospital (n = 4)	Overall (n = 134)
**Staff and guidelines, x̄ (SD)**	0.8 (6.5)	4.4 (14.4)	3.8 (13.6)	25.0 (28.9)	3.4 (12.6)
Trained human resource	1 (1.7)	2 (4.4)	1 (3.8)	1 (25.0)	5 (3.7)
PEN guidelines	0 (0.0)	2 (4.4)	1 (3.8)	1 (25.0)	4 (3.0)
**Equipment x̄ (SD)**	86.4 (17.6)	99.3 (5.0)	100 (0.0)	100 (0.0)	93.8 (13.7)
Blood pressure gauge	59 (100.0)	45 (100.0)	26 (100.0)	4 (100.0)	134 (100.0)
Weight measuring equipment	58 (98.3)	45 (100.0)	26 (100.0)	4 (100.0)	133 (99.3)
Height measuring equipment	36 (61.0)	44 (97.8)	26 (100.0)	4 (100.0)	110 (82.1)
**Essential medicines**	53.1 (27.8)	33.3 (0.0)	65.4 (25.8)	66.7 (27.2)	49.3 (25.1)
Thiazide	30 (50.8)	0 (0.0)	17 (65.4)	3 (75.0)	50 (37.3)
Atenolol	12 (20.3)	0 (0.0)	8 (30.8)	1 (25.0)	21 (15.7)
Calcium channel blockers	52 (88.1)	45 (100.0)	26 (100.0)	4 (100.0)	127 (94.8)
**Hypertension service readiness, x̄ (SD)**	46.8 (11.7)	45.7 (5.1)	56.4 (9.8)	63.9 (17.3)	48.8 (10.8)

IQR – interquartile range, MD – median, SD – standard deviation, x̄ – mean

*Values are presented as n (%) unless specified otherwise.

The effects of this lack of training and guidelines were reflected in the qualitative interviews. Participants shared their concerns regarding provider knowledge and subsequent trust:

The doctors do not understand our exact situation or provide information to us. The trust that we have for the doctors, but their response undermines this and does not let us build the trust. The doctor should have provided the information with confidence. When I go back and forth with the doctor asking questions, then they change their statement. So, I now doubt their information. *– (FGD, marginalised urban community, male)*

The lack of confidence in the advice given by health professionals was coupled with a consistent perception of hypertension and diabetes as a common health problem, with most participants reporting at least one household member with these conditions. This led to a belief that these conditions were a natural part of the ageing process:

I was detected with hypertension, but I do not worry much as many of the people around me have the problem. You cannot do anything; at a certain age you will have it. *– (FGD, adult male)*

Participants referred to diabetes as ‘chinirog’ or ‘sugar’, and ‘pressure’ or ‘high blood pressure’ for hypertension. Few participants had detailed knowledge of symptoms, when to go for screening, or the complications and long-term health implications of either condition. For those who did not know their status, it was common for participants to explain that they did not want to seek a diagnosis and know if they had these conditions due to a fear of associated cost and of becoming ‘addicted’ to the medicine:

People say that we should not take the medicines for high blood pressure as it is common, once we start the course, then we will be addicted to the medicine. *– (IDI, adult male with hypertension)*

The lack of trust and appropriate advice from health professionals, coupled with fears of the implications, particularly financial, of having a long-term condition, combined to undermine the willingness of marginalised urban communities to seek health services.

### Management of hypertension and diabetes

Once diagnosed, our quantitative and qualitative findings highlighted the challenges patients faced in managing their hypertension and diabetes within the healthcare system. There was a low overall readiness to manage hypertension (x̄ = 48.8; SD = 10.8) and diabetes (x̄ = 61; SD = 14.7) (**Table 1**, **Table 2**). Primary care, both public and private, was less prepared than hospitals, but all scores were low, particularly for hypertension. All facilities had higher scores for the availability of equipment. Public facilities and private hospitals scored highly on the availability of essential medication for hypertension and diabetes, particularly metformin for diabetes, but limited availability of essential medications for hypertension at both public and private primary care clinics (**Table 2**).

These results were reflected in the qualitative interviews with healthcare providers from public primary care facilities, who acknowledged that they were not offering NCD services as per the Government’s NCD protocol. This was further worsened by the frequent transfer of health providers within the public system:

Due to frequent transfer of staff, including the contractual staff, this adds further vulnerability, as after receiving the training, they leave the facility, and this affects the service delivery. They even take away the guideline, and there is no institutionalisation of the training they received. *– (KII, health facility in-charge, public primary care)*

These challenges, particularly the limited ability of health providers to explain the role of medication in long-term care, led to confusion among patients on how to manage their conditions. Despite gaps in provider knowledge and practices, there were some community participants, mainly younger female participants, who still advocated the importance of taking medicines only after proper diagnosis and consultation with the health service provider:

People with high blood pressure and sugar (diabetes) should take medicines by following proper diagnosis and medical consultation. *– (FGD, young adult female)*

However, for many older patients, there was often a resistance to taking medication for an indefinite period, and few had received any appropriate advice on self-management of their conditions. This meant that if there was no immediate improvement, patients were then sceptical that the medication would work at all and found other explanations for the condition:

If there is a health problem and if it does not get better despite taking medicine, then people think that it may be due to the evil eye, and they might then take ‘fukna’ (spells to cure the effects of evil spirits). Only if the case is too serious, then a family member would take them to the hospital. *– (FGD, female health volunteer)*

Given this limited confidence in allopathic medicine, herbal medicines were also identified as a popular alternative, particularly amongst older patients:

Initially, I was on allopathic medication and one day, my friend suggested I take the leaf of Malabur nut (asuro). I have given the leaf to my mother as well. Now, looking at its effect, I regret that I did not take the leaf earlier. It is ayurvedic and it is better to use. *– (IDI, hypertensive male, 60 years)*

He used to take 18 injections per day because of sugar. In one place, he took some white flowers, and then he got rid of diabetes. *– (IDI, male, 60 years)*

For some, concerns about the effectiveness of the medication meant they emphasised improving their diet and using practices like yoga to support the management of their condition rather than taking medication:

I do not take medicine because I believe eating good food will be enough. Medicine is not effective. *– (IDI, female with diabetes)*

Several healthcare providers commented on the challenges of self-management for long-term conditions such as hypertension and diabetes in the urban area. In rural areas, patients, particularly the elderly, were more likely to have social support through extended families and community structures than in the city:

If you look at the social capital in rural health system like social relationship, social ties, social infrastructure, it is strong and that enables system to deliver services but it is not in urban areas. *– (KII, healthcare provider)*

The limited training and availability of guidelines within primary care, combined with the lack of wider social support, resulted in limited awareness among patients, particularly older patients, about how to manage their condition effectively over the long term. The limited availability of prescribed medicines undermined patients’ willingness to manage their condition within primary care facilities and instead supported their decision to either use herbal alternatives or buy the required medicines at pharmacies.

### Preventive behaviour

In contrast, levels of awareness of the main risk factors of diabetes and hypertension were generally good among community members. Many associated the onset and worsening of both conditions with the consumption of food high in sugar, fats and oils, a high salt intake, physical inactivity, and smoking and tobacco use.

However, interview and focus group participants frequently explained that changing behaviours in the urban context was challenging. Financial constraints were highlighted, with many explaining that healthy food like fruit, fresh vegetables, and diverse staple foods were not an option they could afford.

A consistent theme, particularly among male participants, was the difficulty in stopping or changing tobacco and alcohol use. Despite knowing the dangers of these behaviours, the pressure of the working and living urban environment frequently undermined any attempts to change practices:

The doctor advised not to do heavy work and not to consume tobacco and alcohol. You can face a stroke anytime. At that time, some fear came over me, and I left tobacco but could not get control over alcohol. But slowly, I again started chewing tobacco. I remember the words of doctors till now. Despite these things, I did not have control. *– (FGD, male)*

The stresses of urban living, both physical and mental, played a role in participants’ dependence on tobacco – both smoking and chewing – as well as alcohol. This was often linked to the lack of sufficient nutritious food and was associated with both hypertension and diabetes.

Here, most of the people are daily wage workers. Their bodies demand energising and nutritious food, but they cannot afford it. So instead, they go to the local pub in the evening for alcohol intake. Now I see them suffering from sugar and pressure. *– (IDI, adult female)*

One male member expressed his dependency on alcohol and tobacco use over his health and particularly highlighted the connection to the stress he faced in his daily life:

I will not lie; I take alcohol before dinner and go to my bed. The doctor had shown me a report where black spots were seen in my lungs and told me it was due to smoking. Since then, I have not smoked. But I have not left alcohol because when I am stressed, I take a sip that gives me pleasure. *– (IDI, male with diabetes and hypertension)*

For women, chewing tobacco was more common than smoking, and several women reported that brief advice alone was not sufficient to help them to change their behaviour:

I have high blood pressure, and I was advised to stop chewing tobacco, but I have not stopped chewing it. *– (IDI, female with hypertension)*

The transect walks were conducted at the end of the working day, and this helped us to observe the community once residents returned from work. Transect walk participants explained that in the evening, the community would become bustling and noisy as the many local bars filled up with mainly male daily wage labourers drinking and smoking after a day’s work.

The challenges of changing behaviour were evident among the participants and seem to have been exacerbated by the urban environments in which they are living. However, none reported any counselling from health professionals or any more substantial support to help initiate and maintain changes. This remains consistent with the lack of training among healthcare workers or the availability of any guidelines found in the health facility assessment (**Table 1****,** **Table 2**).

### Accessing care

We highlighted how participants faced financial (*i.e.* opportunity costs and fear of long-term costs of medication), temporal (*i.e.* opening hours and waiting times), and relational (*i.e.* lack of trust in untrained providers and availability of equipment and medicines) barriers to public primary care. Understanding the extent to which the location of services influenced access to care was explored primarily through our mapping of facilities, but also combined with our facility assessment and qualitative work to gain deeper insights.

**Figure 1:**
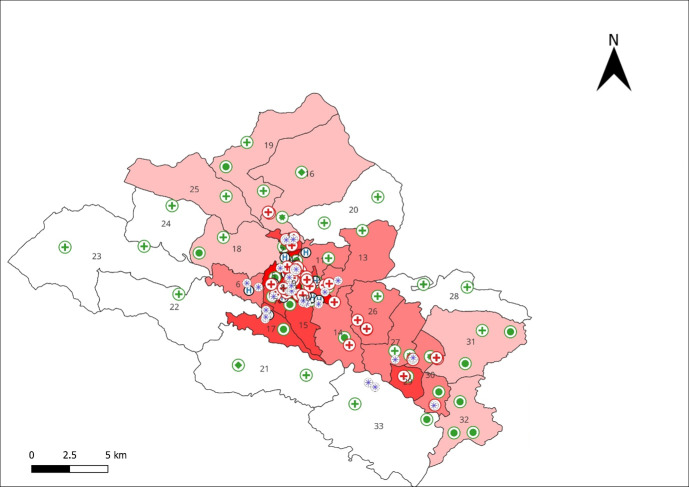
Distribution of health facilities according to population density in Pokhara Metropolitan City.

We identified a total of 660 facilities operational within PMC, and when compared with population density (**Figure 1**), we found that private facilities are more available in densely populated areas, which are characterised by informal settlements and lower-income communities, while most public primary care facilities lie in less dense peri-urban areas with limited employment opportunities.

**Figure 2:**
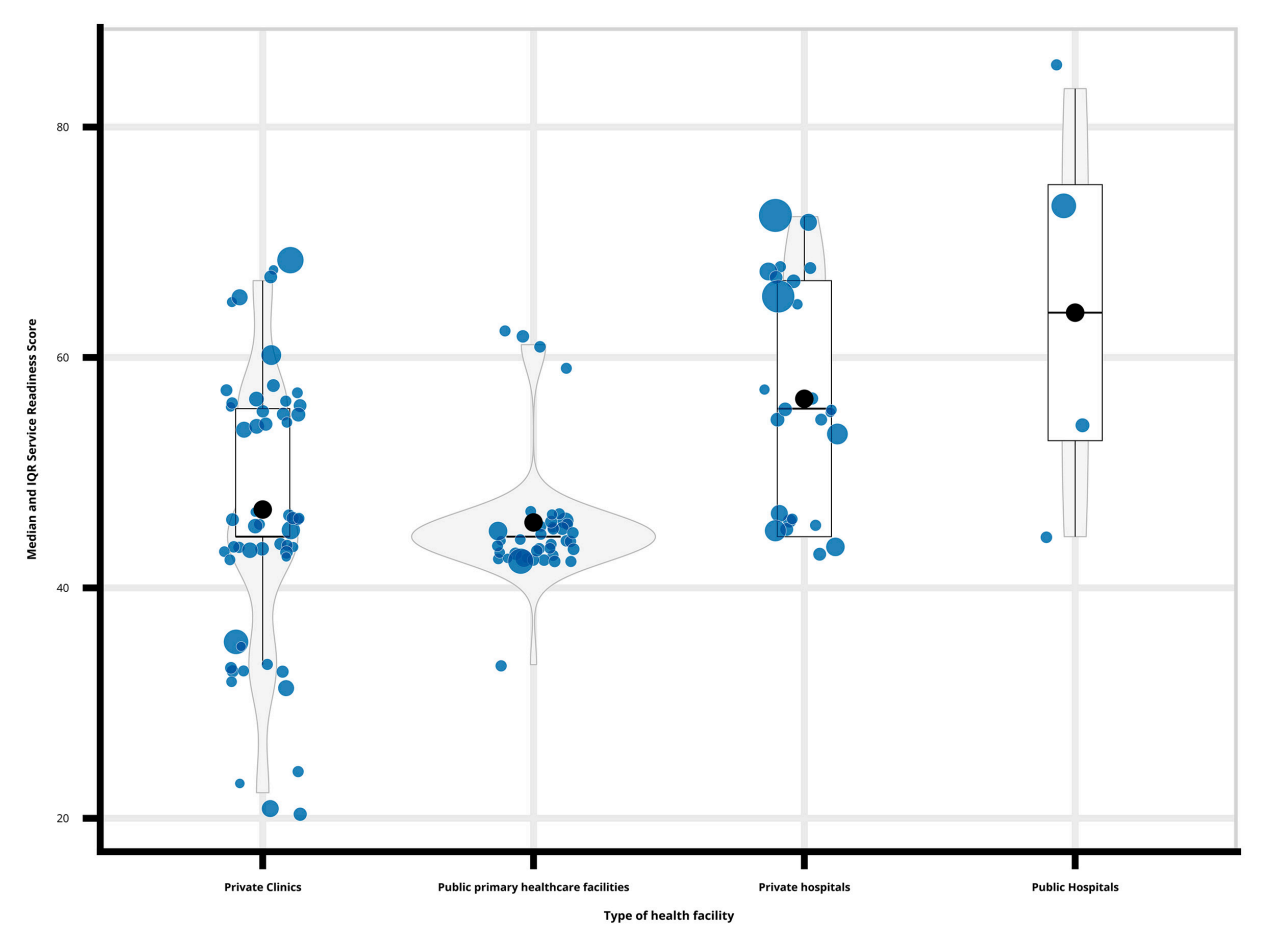
Readiness of hypertension services and patient flow in public and private facilities. IQR – interquartile range.

As suggested by the mapping exercise, private clinics are in the majority and show large volumes of both hypertension and diabetes patients. However, public primary care had a more consistent readiness score than private clinics, private, and public hospitals (**Figure 2**).

**Figure 3:**
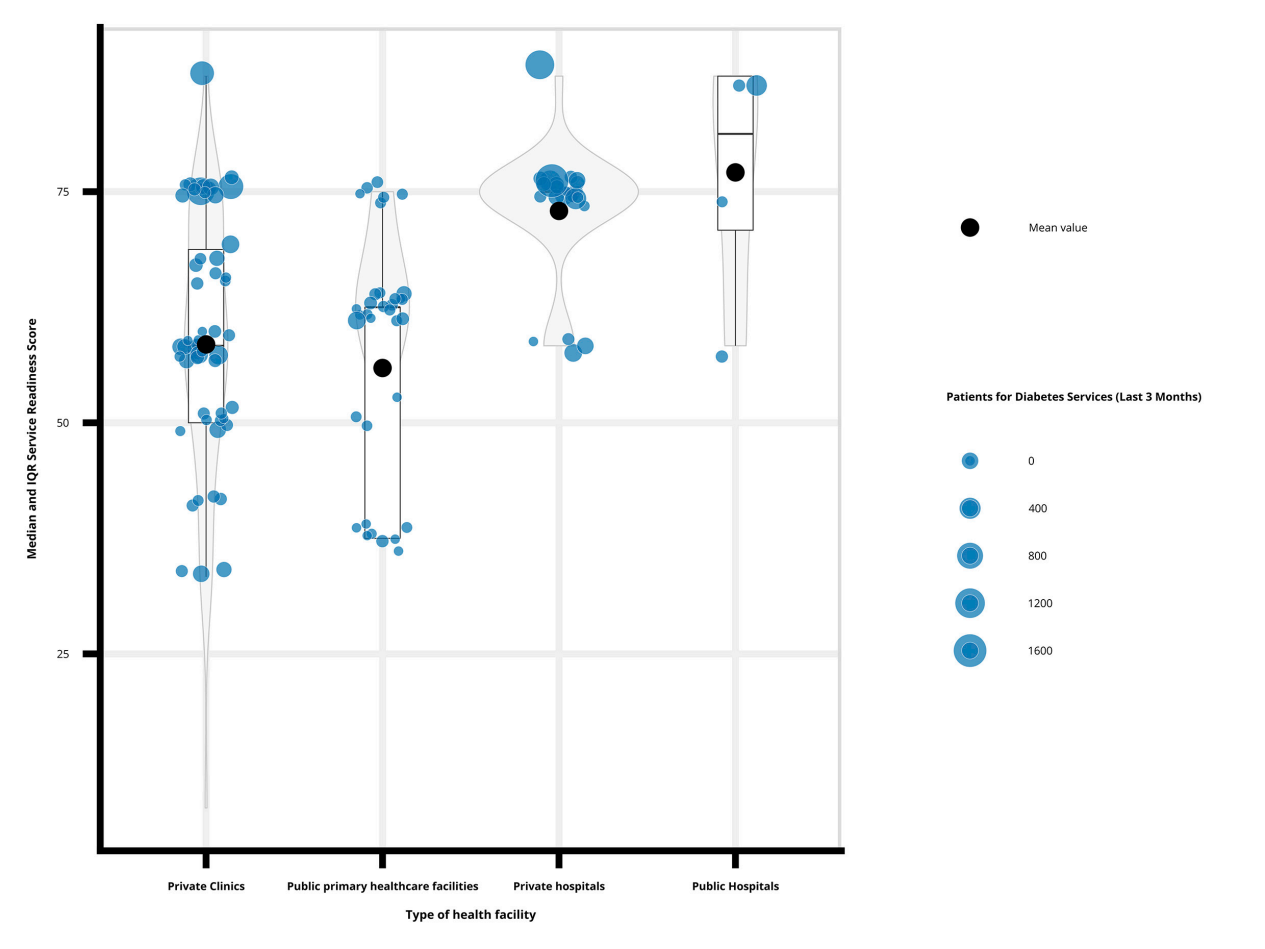
Readiness of diabetes services and patient flow in public and private facilities. IQR – interquartile range.

Similarly, for diabetes, private hospitals and clinics were the most numerous and would also appear to be accessible, as they saw the highest patient numbers (**Figure 3**). However, this data does not indicate the socioeconomic status of patients, and given the high out-of-pocket costs of these providers, these patients are likely wealthier. The overall readiness score among the private clinics showed a wide variation in quality scores. The public primary care clinics showed greater consistency in readiness, even though this was not as high as either the public or private hospitals. Despite this more consistent readiness, public primary care still saw lower patient numbers compared to the private clinics and hospitals. This is consistent with our qualitative findings, which highlight the use of hospitals when conditions worsen, but not the regular use of primary care to manage their condition. The combination of our findings highlights both the limited availability and accessibility of public primary care, with the lack of availability of medication, restrictive opening hours, and limited trust in provider knowledge also influencing this limited use of primary care.

The PMC officials frequently commented on the challenges of providing adequate primary care for the growing city population. The reliance on hospitals to provide services that could be delivered at the primary care level was well recognised. Many raised concerns about the lack of community-based services where strong patient-provider relationships could support the management of diabetes and hypertension, particularly support to understand their condition, medication and lifestyle improvements:

The urban health system is not prepared to deliver services to poor people as in rural areas. Though there are big hospitals, they are crowded and do not focus on public health services. There are no adequate health centres to offer services in densely populated cities like Pokhara. *– (KII, official at health system level)*

The participatory and qualitative findings shed light on the response of urban communities to these challenges of finding adequate care. During the ranking exercise, all six focus groups identified pharmacies (referred to as medical shops or medical clinics) as the most preferred first point of contact due to their proximity, affordability, acceptability, and the quality of information provided. Traditional healers, public health facilities, and private health facilities were also sought for care depending on the perceived seriousness of the health problems.

Pharmacies were clearly seen as ‘part of the community’, with local owners familiar within the community, making them approachable and friendly, allowing customers to have medicines on credit or out-of-hours. One of the participants even described her local pharmacist as a magician. According to her, he provided a ‘*jadu ko goli*’ (*i.e.* magic tablet) to cure her ailment; others in the focus group and interviews agreed and added similar anecdotes:

People usually get medicines from medical shops near their homes when they are sick. If they visit the hospital, they have to follow the required procedures, such as staying in the queue, waiting for the lab results, and going for a further checkup the next day. People must make multiple visits (2–3 days) even for a minor health issue. *– (IDI, male)*

The long working hours of many low-income urban residents and the opportunity cost of missing a day’s income were a particular factor in influencing preference for pharmacies over other providers:

Pharmacies open in the early morning hours and late evening hours, and for people who work as daily wage labourers, they cannot seek services in daytime hours, as they might lose their earnings. *– (KII, male healthcare provider at public health facility)*

Some of the participants, particularly females, reported using local health posts (*i.e.* the lowest level of public primary care) for common ailments, but tended to visit pharmacies to seek treatment when they felt that they had a more severe illness, such as diabetes. Across all the qualitative data sources, participants questioned the rationale for visiting public health facilities. The city officials and healthcare providers also acknowledged this fact:

In a government hospital, the doctor comes very late. People sometimes take their patients to other private hospitals since the doctor came very late. *– (IDI, male, 60 years)*

Given the cost of care for diabetes and hypertension care as long-term conditions and the predominance of use of private providers and pharmacies, we explored strategies to cover the costs of accessing care by low-income households. Health insurance is one option; however, the qualitative interviews highlighted the limited uptake, with many mentioning that they could not afford the premium payments, while others were either not aware of it or did not see any benefit:

I have to pay NPR 3500 (~ USD 25) annually to enrol in the insurance, whether I take the service or not. With that money, I can buy two sacks of rice. *– (FGD, adult female)*

Healthcare providers and city officials concurred with this view, despite local schemes to encourage community members to sign up to the health insurance programme:

The insurance is mostly utilised by middle-class people rather than the urban poor population. Some wards are offering free enrolment of poor households based on recommendations from local representatives. But still, I hardly see such people utilising it. *– (KII, healthcare provider)*

### Population and planning for rising NCDs

Our initial engagements with city actors highlighted a fundamental lack of data to quantify the size and distribution of the urban population, particularly low-income households. Instead, city officials and health system actors focused on apparently easy-to-identify ‘slum areas’. Our participatory methods frequently concurred with this characterisation; however, they also showed that this binary classification was insufficient in capturing the heterogeneity within these ‘slum areas’. During the transect walk, while we observed houses with crumbling walls, sagging, corrugated roofs, narrow walkways, and limited drainage or drinking water, the opinions of community members on what constituted a poor household differed significantly. Community participants frequently cited individuals with white-collar jobs residing in the slum areas, and low-income households clustered in parts of the city not considered slum areas.

Most community participants agreed that occupation or means of livelihood was a more accurate way of assessing the level of poverty than residence location alone. Those working as daily wage labourers in construction sites or as porters, as well as the disabled and the elderly relying on others or governmental allowance for their survival, were seen as particularly marginalised. Some participants felt a new category of the ‘ultra poor’ was required to capture these distinctions. Poverty was not seen as only a characteristic of those in the informal sector, with workers in the formal sector also considered poor, particularly low-paid salary jobs like security guards and office assistants, particularly when their families had no other sources of income:

There lives an elderly woman in a very well-off family who also owns a car. But the woman does not get any financial support from her son, and she cannot even afford the minimum healthcare services. Does that woman fall under well off or poor? *– (female community health volunteer during social mapping)*

Community participants were clear that only identifying poor households was not enough, and poor individuals were likely to exist within better-off families. There was unanimous agreement among the stakeholders that there cannot be one precise definition of what it means to be poor in the urban context.

Across all interviews with health system actors and healthcare providers, there was agreement that there was no formal mechanism to identify and target urban poor communities. This is despite a budget allocation within health services for poor and marginalised sections of the community. Participants reported that locally elected representatives would personally recommend an individual as poor and deserving of targeted services, with no transparent and defined mechanism:

Despite we say equity in our plan and policies, our existing healthcare service takes a blanket approach, and we do not have any customised strategy to reach out to the poor and vulnerable people with the services. *– (KII, official at health system level)*

While our qualitative and participatory methods identified the challenges with data and information to understand the urban population and identify poor individuals and households, our health facility assessments highlighted challenges in using routine health service data to understand the population's need for and plan hypertension and diabetes services. Over 20% of facilities did not record patient data, and less than half had a focal person responsible for recording data. All public hospitals (100%), almost all peripheral facilities (97.8%), and private hospitals (88.5%) recorded patient data. However, only a little over half the clinics practised recording, and most of these were using digital recording systems (67.9%). Approximately one-third of public primary care clinics had digital recording systems, although less than half had a focal person responsible for recording data. Where data on NCDs were recorded within public facilities, these were aggregated to calculate key NCD indicators such as the number of patients screened, diagnosed, and treated for hypertension and/or diabetes.

## DISCUSSION

With this mixed-methods study, we provide critical insights into the readiness of the health facilities to manage hypertension and diabetes among the urban poor in a secondary city in Nepal. The findings reveal significant gaps in service readiness in both private and public PHC facilities, including inadequate infrastructure, insufficient medical supplies, and particularly a lack of trained healthcare providers with access to guidelines. This aligns with previous studies conducted in Nepal [40,41] and other LMICs [42-46]. This is particularly concerning given the rising prevalence of hypertension (28.5%) and diabetes (8.5%) in Nepal [47,48]. Our qualitative findings highlight the challenges posed by inadequate training and high staff turnover, with providers feeling ill-prepared to manage NCDs, echoing findings from other LMICs, including South Asian countries, where limited capacity has been a persistent barrier to NCD care [45,46].

These challenges are compounded by systemic barriers that limit access to care for the urban poor, particularly the dominance of private providers in areas of high population density, further exacerbating health inequities. This aligns with evidence across LMICs that informal and formal private providers, particularly pharmacies, are frequently the first point of contact for low-income urban residents [49,50]. While there is evidence that urban residents may bypass local providers for public hospitals, particularly when they are concerned about quality [51], our findings highlight that for hypertension and diabetes services, concerns about waiting times, limited training, and readiness – as supported by our facility assessments – may be undermining use of public facilities. Given the changing disease burden and limitations in public primary care, the scope of pharmacy practice in Nepal is no longer limited to dispensing the drugs as traditionally allowed within pharmaceutical policies. Increasingly, the pharmacies have expanded their offer to include a wide range of healthcare services, including those for hypertension and diabetes [39].

This expansion of private providers, including pharmacies, in rapidly urbanising LMICs is gaining increasing attention in the health systems literature [52,53]. Studies have found that private pharmacies can support the prevention and management of hypertension and diabetes; however, most are feasibility studies and are often short-term projects not designed for sustainability within the primary care system. Studies that have explored knowledge of private providers in urban poor environments on hypertension and diabetes [54] and pharmacy practices in relation to providing diabetes and hypertension services in these contexts have found poor knowledge and practice, including in Nepal [39].

Within the current policy in Nepal, the role of pharmacies is limited to dispensing medication based on a prescription provided by a physician. Yet, most of the pharmacies in Nepal are operated by trained paramedics with recognised credentials [39,55]. Following additional NCD training in accordance with the Government’s Multi-sectoral Action Plan for NCD Prevention and Control (2014–2020), the same paramedics also operate in public health facilities, delivering basic NCD care services. With a similar level of additional training, pharmacies could potentially deliver screening, counselling, and referral for NCDs. Given that pharmacies are clearly the first point of contact for many urbanites, particularly the most marginalised, there is a need for a policy change to reflect the reality of this expanding role. However, any change must also address the multiple challenges that have been identified with common practice within pharmacies in LMICs, including poor history-taking practice, failure to refer patients requiring medical attention, illegal sale of prescription-only medicines without prescriptions, irrational use of drugs, and inadequate information or counselling provided to patients [56]. Implementation research to evaluate system-wide responses to establish and maintain service linkages between pharmacies and the public health system is needed. Identifying how to incentivise pharmacies and other private providers to maintain quality and report patient data on NCDs is currently lacking within the routine health information needs further exploration.

We highlighted that marginalised urban poor individuals and households, particularly daily wage labourers, face challenges in adopting healthy behaviours due to their socioeconomic status and wider environment within the urban context. We identified high alcohol and tobacco use among low-income men, consistent with other studies [57,58]. The increased exposure of urban residents to unhealthy foods and perceptions about the benefit of alcohol to fuel manual daily-wage labour, access to tobacco products, and the high cost of fruit and vegetables is coupled with the lack of training of health professionals to support patients with diabetes and/or hypertension to adopt a healthy lifestyle. This is further aggravated by the lack of focus on prevention or a cadre of community health workers or volunteers in urban areas to propagate these messages, particularly in the face of increased advertising within the commercial, urban environment.

We elucidate the complex perceptions of diabetes and hypertension, and due to their high prevalence, many see these conditions as an inevitable part of ageing, a phenomenon found in many contexts and a recognised factor in delaying diagnosis and treatment [59,60]. The structural vulnerabilities of such urban communities were clearly in evidence through their reliance on the informal sector and the resultant daily-wage labour [61]. This undermined the ability to access free public primary care and to sustain the costs of long-term medication for a chronic condition. Similar structural vulnerabilities have been noted as barriers to hypertension treatment in Nepal’s capital, Kathmandu [62].

With trust and familiarity with herbal and other traditional medicines, many see non-allopathic treatments as a logical choice, a practice seen in similar settings [62]. However, participants also highlighted the use of higher levels of formal care, particularly hospitals, when they considered symptoms to be severe. This reflects a thoughtful syncretic use of health providers, as has been found among NCD patients in informal settlements in Sierra Leone [63]. Similarly, people’s awareness of hypertension, experience of symptoms, and the process of their diagnosis and care have been shown to influence people’s use of self-care and the plurality of providers through their own ‘itineraries of care’ in the Philippines [64].

We also highlight the complexity of identifying and understanding urban poverty, and particularly who may be most vulnerable to poor health and healthcare access. While the initial qualitative findings with decision-makers suggested identifying slum areas would be relatively straightforward, our participatory work highlighted the complexity of poverty and vulnerability within the wards studied. This is reflected in the challenges of defining urban poverty [65], particularly considering poverty as an absolute measurement or poverty line [66], and supports approaches such as the multi-source data poverty index [67] to address the complex interplay of socioeconomic factors. However, in a resource-limited setting such as Pokhara, the multiple data sources required for such a measure are rarely available. The city, like many others in LMICs, also faces considerable challenges in identifying the total population of the city, given high levels of migration and transience within the city. While such qualitative and participatory methods do not provide a comparable approach to quantify urban poverty or target services, community-based participatory methods [36] were valuable to understand the nuances of urban poverty and to purposively sample and reach marginalised urban dwellers [65,68]. Improving methods for identifying and understanding urban poverty are key to helping local governments plan to meet the needs of their growing populations and changing disease burdens. Drawing on detailed qualitative and participatory work can provide a valuable local ‘sense-check’ against national poverty measures that may not adequately reflect the realities and dimensions of urban poverty.

### Limitations and strengths

The mixed-methods design allowed us to explore the impact of limited diabetes and hypertension service readiness on the health-seeking behaviour of urban poor communities. We conducted the health facility assessment and the qualitative analyses within the same period, which ensured the alignment between the reflections of community members and the readiness assessed in the health facilities. However, participants did not restrict their contributions to the period of the health facility assessment, and this should be considered when interpreting the findings. We based our health facility readiness assessment on our observations and self-reports from the facility staff, which may have led to an overestimation in the descriptions of training received due to social desirability, or an overstatement of the actual functional capacity of the equipment. Additionally, we did not assess provider knowledge, quality control, calibration, accuracy or availability of consumables.

Our qualitative analyses drew on participatory engagement with communities to identify urban poor participants for interviews. This proved a strength in understanding the complexities of urban poverty. However, due to social and gender norms within the communities, consent to participate in interviews and FGDs may still have privileged the more socially included. For example, despite our attempts to ensure accessibility and to include people with disabilities, there were no such participants in our sample. We found that holding separate male and female focus groups was one strategy that enabled women in the community to speak openly about their experiences and health seeking behaviour, including the influence of patriarchal norms on their lives. The use of individual interviews as well as focus groups and participatory methods was helpful in hearing perspectives of marginalised individuals, which may potentially be overlooked in the group-based data collection methods. It should also be noted that, while based on detailed data collection using multiple methods, we generated our qualitative findings from two communities within one city in Nepal. Given that Pokhara shares many characteristics of rapid urbanisation and economic growth with other cities in Nepal, we believe many elements of our findings are transferable. However, findings may be more relevant to cities within the mid-hills zone of Nepal.

We developed the composite scores for hypertension and diabetes readiness using the relevant indicators from WHO’s SARA tool [32], which is in line with the factors seen as essential within GoN NCD protocols. We believe this is a strength in our approach; however, it should be noted that individual items have been given equal weighting within the composite score.

## CONCLUSIONS

Despite the diversity of healthcare providers in urban areas, the system showed poor readiness to manage hypertension and diabetes, particularly for the urban poor. The consequences of these service limitations are reflected in the high-risk behaviours observed among the urban poor, including unwillingness to seek diagnosis for hypertension and diabetes and limited willingness or ability to adhere to long-term, costly medications. In Nepal’s federalised system, decentralised health system strengthening remains at an early stage, creating an opportunity to explore how private providers, such as pharmacies, can be better integrated into primary care to improve access to NCD services. Such strategies must be grounded in local realities and account for the plurality of providers operating in metropolitan cities like Pokhara. Addressing urban poverty and developing financing mechanisms that facilitate access to NCD care for vulnerable urban populations should be a priority, yet this remains an underutilised area. Without context-specific health system reforms, service provision is likely to remain fragmented, resulting in inadequate access to quality NCD care for the urban poor.

## Additional material


Online Supplementary Document

## References

[1] World Health Organization. Global status report on non-communicable diseases. Geneva, Switzerland: World Health Organization; 2010. Available: https://iris.who.int/server/api/core/bitstreams/625932a8-bb29-425b-9ce2-f10cb8cd8292/content. Accessed: 9 August 2025.

[2] United Nations Human Settlements Programme. The challenge of slums: global report on human settlements 2003. London: Earthscan Publications Ltd; 2003. Available: https://unhabitat.org/sites/default/files/download-manager-files/The%20Challenge%20of%20Slums%20-%20Global%20Report%20on%20Human%20Settlements%202003.pdf. Accessed: 23 June 2026.

[3] Allender S Wickramasinghe K Goldacre M Matthews D Katulanda P Quantifying urbanization as a risk factor for noncommunicable disease. J Urban Health. 2011;88:906–18. 10.1007/s11524-011-9586-1 21638117 PMC3191205

[4] Uthman OA Ayorinde A Oyebode O Sartori J Gill P Lilford RJ Global prevalence and trends in hypertension and type 2 diabetes mellitus among slum residents: A systematic review and meta-analysis. BMJ Open. 2022;12:e052393. 10.1136/bmjopen-2021-052393 35210339 PMC8883228

[5] Olufayo OE Asowata OJ Okekunle AP Akpa OM Hypertension burden and associated risk factors among people from the slums in a developing country: evidence from the COMBAT-CVD study. J Hum Hypertens. 2025;39:755–63. 10.1038/s41371-025-01057-x 40858979 PMC12592212

[6] Mistry SK Hossain MB Parvez M Das Gupta R Arora A Prevalence and determinants of hypertension among urban slum dwellers in Bangladesh. BMC Public Health. 2022;22:2063. 10.1186/s12889-022-14456-3 36368965 PMC9650885

[7] Ezeh A Oyebode O Satterthwaite D Chen YF Ndugwa R Sartori J The history, geography, and sociology of slums and the health problems of people who live in slums. Lancet. 2017;389:547–58. 10.1016/S0140-6736(16)31650-6 27760703

[8] Juma K, Juma PA, Shumba C, Otieno P, Asiki G. Non-Communicable Diseases and Urbanization in African Cities: A Narrative Review. In: Anugwom EE, Awofeso N, editors. Public Health in Developing Countries - Challenges and Opportunities. London, UK: IntechOpen; 2020. p. 47–66.

[9] French Agricultural Research Centre for International Development, European Union, Centre de coopération internationale en recherche agronomique pour le développement. Food Systems Profile - Nepal. Montpellier, France: French Agricultural Research Centre for International Development; 2022. Available: https://openknowledge.fao.org/items/52fb1af8-8ba3-4b62-b08a-4a6b201ee527. Accessed: 9 August 2025.

[10] Nsabimana P Sombié OO Pauwels NS Boynito WG Tariku EZ Vasanthakaalam H Association between urbanization and metabolic syndrome in low- and middle-income countries: A systematic review and meta-analysis. Nutr Metab Cardiovasc Dis. 2024;34:235–50. 10.1016/j.numecd.2023.07.040 38182494

[11] Aryal KK Mehata S Neupane S Vaidya A Dhimal M Dhakal P The burden and determinants of non communicable diseases risk factors in Nepal: Findings from a nationwide STEPS survey. PLoS One. 2015;10:e0134834. 10.1371/journal.pone.0134834 26244512 PMC4526223

[12] Tee GH Aris T Rarick J Irimie S Social determinants of health and Tobacco use in five low - and middle-income countries - results from the Global Adult Tobacco Survey (GATS), 2011-2012. Asian Pac J Cancer Prev. 2016;17:1269–76. 10.7314/APJCP.2016.17.3.1269 27039759

[13] Allen LN Townsend N Williams J Mikkelsen B Roberts N Wickramasinghe K Socioeconomic status and alcohol use in low- and lower-middle income countries: A systematic review. Alcohol. 2018;70:23–31. 10.1016/j.alcohol.2017.12.002 29723827

[14] Kakchapati S Neupane R Baral KS Shrestha G Joshi D Dawkins B Social determinants and risk factors associated with non-communicable diseases among urban population in Nepal: A comparative study of poor, middle and rich wealth categories of urban population using STEPS survey. PLoS One. 2025;20:e0307622. 10.1371/journal.pone.0307622 40367119 PMC12077703

[15] Lilford RJ Daniels B McPake B Bhutta ZA Mash R Griffiths F Policy and service delivery proposals to improve primary care services in low-income and middle-income country cities. Lancet Glob Health. 2025;13:e954–66. 10.1016/S2214-109X(24)00536-9 40288403

[16] Prinja S Purohit N Kaur N Rajapaksa L Sarker M Zaidi R The state of primary health care in south Asia. Lancet Glob Health. 2024;12:e1693–705. 10.1016/S2214-109X(24)00119-0 39178880

[17] Sriram V Yilmaz V Kaur S Andres C Cheng M Meessen B The role of private healthcare sector actors in health service delivery and financing policy processes in low-and middle-income countries: a scoping review. BMJ Glob Health. 2024;8:e013408. 10.1136/bmjgh-2023-013408 38316466 PMC11077349

[18] Saito E Gilmour S Yoneoka D Gautam GS Rahman MM Shrestha PK Inequality and inequity in healthcare utilization in urban Nepal: A cross-sectional observational study. Health Policy Plan. 2016;31:817–24. 10.1093/heapol/czv137 26856362 PMC4977425

[19] Khanal A. GC S, Dahal I, Mishra S, GC VS, Wasti SP, et al. Hypertension care cascade in Nepal: findings from Nepal Demographic and Health Survey 2022. medRxiv: 25323662v1 [preprint]. 2025. Available: http://medrxiv.org/lookup/doi/10.1101/2025.03.10.25323662 doi:10.1101/2025.03.10.25323662. Accessed: 9 August 2025.

[20] Mistry SK Hossain MB Parvez M Das Gupta R Arora A Prevalence and determinants of hypertension among urban slum dwellers in Bangladesh. BMC Public Health. 2022;22:2063. 10.1186/s12889-022-14456-3 36368965 PMC9650885

[21] NCD Risk Factor Collaboration (NCD-RisC) Worldwide trends in diabetes prevalence and treatment from 1990 to 2022: a pooled analysis of 1108 population-representative studies with 141 million participants. Lancet. 2024;404:2077–93. 10.1016/S0140-6736(24)02317-1 39549716 PMC7616842

[22] Schutte AE Srinivasapura Venkateshmurthy N Mohan S Prabhakaran D Hypertension in Low- And Middle-Income Countries. Circ Res. 2021;128:808–26. 10.1161/CIRCRESAHA.120.318729 33793340 PMC8091106

[23] Abbafati C Abbas KM Abbasi-Kangevari M Abd-Allah F Abdelalim A Abdollahi M Global burden of 369 diseases and injuries in 204 countries and territories, 1990–2019: a systematic analysis for the Global Burden of Disease Study 2019. Lancet. 2020;396:1204–22. 10.1016/S0140-6736(20)30925-9 33069326 PMC7567026

[24] GBD 2019 Diseases and Injuries Collaborators Diabetes risk and provision of diabetes prevention activities in 44 low-income and middle-income countries: a cross-sectional analysis of nationally representative, individual-level survey data. Lancet Glob Health. 2023;11:e1576–86. 10.1016/S2214-109X(23)00348-0 37734801 PMC10560068

[25] Ndubuisi NE Noncommunicable Diseases Prevention In Low- and Middle-Income Countries: An Overview of Health in All Policies (HiAP). Inquiry. 2021;58:46958020927885. 10.1177/0046958020927885 34420412 PMC8385577

[26] Guetterman TC Fetters MD Creswell JW Integrating quantitative and qualitative results in health science mixed methods research through joint displays. Ann Fam Med. 2015;13:554–61. 10.1370/afm.1865 26553895 PMC4639381

[27] Poudel KR Origin and Growth of Urban Centers: A Study of Pokhara Metropolitan City, Nepal. The Himalayan Geographers. 2025;15:45–63. 10.3126/thg.v15i1.81412

[28] Governance and Social Development Resource Centre. Urbanisation and urban growth in Nepal. London, UK: Governance and Social Development Resource Centre; 2015. Available: https://gsdrc.org/wp-content/uploads/2015/11/HDQ1294.pdf. Accessed: 9 August 2025.

[29] International Organization for Migration. Migration Profile of Gandaki Province, Nepal 2023. Kathmandu, Nepal: International Organization for Migration; 2024. Available: https://publications.iom.int/system/files/pdf/pub2023-056-el-mp-gandaki-province_0.pdf. Accessed: 9 August 2025.

[30] Baral MP Estimation of Internal Migration in Gandaki Province Using Indirect Techniques. Kalika Journal of Multidisciplinary Studies. 2021;3:11–9. 10.3126/kjms.v3i1.48180

[31] United Nations Human Settlements Programme. Urban Indicators Database. 2022. Available: https://data.unhabitat.org/pages/housing-slums-and-informal-settlements. Accessed: 9 August 2025.

[32] World Health Organization. Service availability and readiness assessment (SARA): an annual monitoring system for service delivery: reference manual. Geneva, Switzerland: World Health Organization; 2013. Available: https://cdn.who.int/media/docs/default-source/service-availability-and-readinessassessment(sara)/sara_reference_manual_chapter3.pdf. Accessed: 9 August 2025.

[33] Bintabara D Ngajilo D Readiness of health facilities for the outpatient management of non-communicable diseases in a low-resource setting: An example from a facility-based cross-sectional survey in Tanzania. BMJ Open. 2020;10:e040908. 10.1136/bmjopen-2020-040908 33177143 PMC7661355

[34] Salauddin M Anee US Barua D Hicks J Islam K Elsey H How prepared are urban primary care facilities to manage hypertension and type 2 diabetes in Dhaka, Bangladesh? A cross-sectional descriptive study of government urban dispensaries and NGO clinics. BMC Prim Care. 2026;27:67. 10.1186/s12875-025-03144-x 41501642 PMC12918133

[35] Ministry of Health and Population. Package of Essential Non Communicable Disease (PEN) Intervention at Primary Health Service Setting. Kathmandu, Nepal: Ministry of Health and Population; 2019. Available: https://edcd.gov.np/uploads/resource/5c39743267983.pdf. Accessed: 9 August 2025.

[36] Kumar S. Methods for community participation: a complete guide for practitioners. Warwickshire, UK: ITDG Publishing; 2002.

[37] Attride-Stirling J Thematic Networks: an analytic tool for qualitative research. Qual Res. 2001;1:385–405. 10.1177/146879410100100307

[38] O’Brien BC Harris IB Beckman TJ Reed DA Cook DA Standards for reporting qualitative research: A synthesis of recommendations. Acad Med. 2014;89:1245–51. 10.1097/ACM.0000000000000388 24979285

[39] Shrestha G Joshi D Sapkota PM Kakchapati S Dawkins B Elsey H A quantitative assessment of current practice in diabetes and hypertension services in pharmacies in urban Nepal. PLoS One. 2025;20:e0328827. 10.1371/journal.pone.0328827 40680079 PMC12273978

[40] Adhikari B Pandey AR Lamichhane B Kc SP Joshi D Regmi S Readiness of health facilities to provide services related to non-communicable diseases in Nepal: evidence from nationally representative Nepal Health Facility Survey 2021. BMJ Open. 2023;13:e072673. 10.1136/bmjopen-2023-072673 37423630 PMC10335515

[41] Sapkota BP Baral KP Berger U Parhofer KG Rehfuess EA Health sector readiness for the prevention and control of non-communicable diseases: A multi-method qualitative assessment in Nepal. PLoS One. 2022;17:e0272361. 10.1371/journal.pone.0272361 36178897 PMC9524672

[42] Seiglie JA Serván-Mori E Begum T Meigs JB Wexler DJ Wirtz VJ Predictors of health facility readiness for diabetes service delivery in low- and middle-income countries: The case of Bangladesh. Diabetes Res Clin Pract. 2020;169:108417. 10.1016/j.diabres.2020.108417 32891691 PMC8092080

[43] Ahmed S Cao Y Wang Z Coates MM Twea P Ma M Service readiness for the management of non-communicable diseases in publicly financed facilities in Malawi: Findings from the 2019 Harmonised Health Facility Assessment census survey. BMJ Open. 2024;14:e072511. 10.1136/bmjopen-2023-072511 38176873 PMC10773330

[44] Singh S Kaul M Rawandale CJ An analysis of health facility services readiness for non-communicable diseases in 8 LMICs in the universal health coverage era. Health Promot Perspect. 2024;14:343–9. 10.34172/hpp.43175 40041736 PMC11873774

[45] Albelbeisi AH Albelbeisi A El Bilbeisi AH Taleb M Takian A Akbari-Sari A Public Sector Capacity to Prevent and Control of Noncommunicable Diseases in Twelve Low- and Middle-Income Countries Based on WHO-PEN Standards: A Systematic Review. Health Serv Insights. 2021;14:1178632920986233. 10.1177/1178632920986233 33597808 PMC7863145

[46] Ahmed SM Krishnan A Karim O Shafique K Naher N Srishti SA Delivering non-communicable disease services through primary health care in selected south Asian countries: are health systems prepared? Lancet Glob Health. 2024;12:e1706–19. 10.1016/S2214-109X(24)00118-9 39178879 PMC11413526

[47] Shrestha DB Budhathoki P Sedhai YR Baniya A Lamichhane S Shahi M Prevalence, awareness, risk factors and control of hypertension in Nepal from 2000 to 2020: A systematic review and meta-analysis: Hypertension in Nepal: A Systematic review and meta-analysis. Public Health Pract (Oxf). 2021;2:100119. 10.1016/j.puhip.2021.100119 36101638 PMC9461174

[48] Shrestha N Mishra SR Ghimire S Gyawali B Mehata S Burden of Diabetes and Prediabetes in Nepal: A Systematic Review and Meta-Analysis. Diabetes Ther. 2020;11:1935–46. 10.1007/s13300-020-00884-0 32712902 PMC7434818

[49] Adams AM Islam R Ahmed T Who serves the urban poor? A geospatial and descriptive analysis of health services in slum settlements in Dhaka, Bangladesh. Health Policy Plan. 2015;30:i32–45. 10.1093/heapol/czu094 25759453 PMC4353891

[50] Watson S Pharmacies in informal settlements: a retrospective, cross-sectional household and health facility survey in four countries. BMC Health Serv Res. 2021;21:945. 10.1186/s12913-021-06937-9 34503501 PMC8431901

[51] Conlan C Cunningham T Watson S Madan J Sfyridis A Sartori J Perceived quality of care and choice of healthcare provider in informal settlements. PLOS Glob Public Health. 2023;3:e0001281. 10.1371/journal.pgph.0001281 36962860 PMC10022014

[52] Adams AM Nambiar D Siddiqi S Alam BB Reddy S Advancing universal health coverage in South Asian cities: A framework. BMJ. 2018;363:k4905. 10.1136/bmj.k4905 30498010 PMC7115914

[53] Afaq S Elsey H Ismail M Islam K Huque R Walker S Unlocking the potential of community pharmacies to address hypertension in South Asia: the COPE-BP programme. J Glob Health. 2026;16:03005. 10.7189/jogh.16.03005 41718000 PMC12922466

[54] Mbachu CO Arize I Obi C Ebenso B Elsey H Onwujekwe O Assessing knowledge of hypertension and diabetes mellitus among informal healthcare providers in urban slums in southeastern Nigeria. Discov Public Health. 2024;21:21. 10.1186/s12982-024-00143-8

[55] Ban B Hodgins S Thapa P Thapa S Joshi D Dhungana A A national survey of private-sector outpatient care of sick infants and young children in Nepal. BMC Health Serv Res. 2020;20:545. 10.1186/s12913-020-05393-1 32546276 PMC7298835

[56] Miller R Goodman C Performance of retail pharmacies in low- and middle-income Asian settings: a systematic review. Health Policy Plan. 2016;31:940–53. 10.1093/heapol/czw007 26962123 PMC4977427

[57] J NirmalaC Dharaneesh S P. A study on addiction pattern among construction workers in a metropolitan city of Southern India. RGUHS Nat J Public Health. 2018;3:13–6.

[58] Panigrahi A Das BC Panigrahi M Tobacco use among daily wage laborers in the city of Bhubaneswar, Odisha, India. J Public Health. 2013;21:57–61. 10.1007/s10389-012-0521-z

[59] Du QH Yang JH Zhang ZC Li SB Liu YQ Li YM Exploring the decision-making experience of elderly diabetes patients regarding their health-seeking behaviour: a descriptive qualitative study. BMJ Open. 2024;14:e087126. 10.1136/bmjopen-2024-087126 39424381 PMC11492961

[60] Yous ML Ganann R Ploeg J Markle-Reid M Northwood M Fisher K Older adults’ experiences and perceived impacts of the Aging, Community and Health Research Unit-Community Partnership Program (ACHRU-CPP) for diabetes self-management in Canada: A qualitative descriptive study. BMJ Open. 2023;13:e068694–10. 10.1136/bmjopen-2022-068694 37019487 PMC10083734

[61] Tan ST Low PTA Howard N Yi H Social capital in the prevention and management of non-communicable diseases among migrants and refugees: A systematic review and meta-ethnography. BMJ Glob Health. 2021;6:e006828. 10.1136/bmjgh-2021-006828 34952855 PMC8710856

[62] Bhandari B Narasimhan P Vaidya A Subedi M Jayasuriya R Barriers and facilitators for treatment and control of high blood pressure among hypertensive patients in Kathmandu, Nepal: a qualitative study informed by COM-B model of behavior change. BMC Public Health. 2021;21:1524. 10.1186/s12889-021-11548-4 34372808 PMC8351340

[63] Conteh A Dean L Wilkinson A Macarthy J Koroma B Theobald S Health seeking by people living with non-communicable diseases in a pluralistic health system: the role of informal healthcare providers. Int J Equity Health. 2025;24:67. 10.1186/s12939-025-02428-z 40069705 PMC11895327

[64] Mendoza JA Lasco G Renedo A Palileo-Villanueva L Seguin M Palafox B (De)constructing ‘therapeutic itineraries’ of hypertension care: A qualitative study in the Philippines. Soc Sci Med. 2022;300:114570. 10.1016/j.socscimed.2021.114570 34802782 PMC7613024

[65] Ensor T Bhattarai R Manandhar S Poudel AN Dhungel R Baral S From rags to riches: Assessing poverty and vulnerability in urban Nepal. PLoS One. 2020;15:e0226646. 10.1371/journal.pone.0226646 32023251 PMC7001899

[66] Boonyabancha S Kerr T How urban poor community leaders define and measure poverty. Environ Urban. 2015;27:637–56. 10.1177/0956247815600945

[67] Niu T Chen Y Yuan Y Measuring urban poverty using multi-source data and a random forest algorithm: A case study in Guangzhou. Sustain Cities Soc. 2020;54:102014. 10.1016/j.scs.2020.102014

[68] Karuga R Kabaria C Chumo I Okoth L Njoroge I Otiso L Voices and challenges of marginalized and vulnerable groups in urban informal settlements in Nairobi, Kenya: building on a spectrum of community-based participatory research approaches. Front Public Health. 2023;11:1–17. 10.3389/fpubh.2023.1175326 38074741 PMC10701261

