# Supplementary material for: What happens when public primary care is ill-prepared to respond to non-communicable diseases: a mixed-method study of diabetes and hypertension care in urban Nepal
Source: J Glob Health. 2026 Jul 10;16:04218. doi: 10.7189/jogh.16.04218 (PMC13348664; doi:10.7189/jogh.16.04218)
Supplement: Online Supplementary Document [file jogh-16-04218-s001.pdf]

Supplement to: Joshi D, Elsey H, Shrestha G, Neupane R, Sapkota PM, Adhikari B, Kakchapati S, Giri S, Arjyal A, Sharma S, Poudel S, Baral SC. What happens when public primary care is ill-prepared to respond to non-communicable diseases: a mixed-method study of diabetes and hypertension care in urban Nepal. J Glob Health. 2026;16:04218.

Table S1: Hypertension readiness score calculations

Staff and Guidelines	T1	Availability of human resources trained/ refreshed (within the last two years) in management of hypertension	No = 0<br>Yes = 1	Staff and Guidelines Readiness Score = $\frac{T1 + T2}{2} \times 100$
	T2	Availability of PEN guidelines	No = 0<br>Yes = 1	
Equipment	T3	Availability of functioning BP set	No = 0<br>Yes = 1	Equipment Readiness Score = $\frac{T3 + T4 + T5}{3} \times 100$
	T4	Availability of weight measuring equipment	No = 0<br>Yes = 1	
	T5	Availability of height measuring equipment	No = 0<br>Yes = 1	
Essential Medicines	T6	Availability of Thiazide	No = 0<br>Yes = 1	Essential Medicines Readiness Score = $\frac{T6 + T7 + T8}{3} \times 100$
	T7	Availability of Atenolol	No = 0<br>Yes = 1	
	T8	Availability of Calcium Channel Blockers	No = 0<br>Yes = 1	
Hypertension Service Readiness = $\frac{Staff\ and\ Guidelines + Equipment + Essential\ Medicines\ Readiness}{3}$				

Table S2: Diabetes readiness score calculations

Staff and Guidelines	T1	Availability of Human Resource trained/ refreshed (within the last two years) in management of diabetes	No = 0<br>Yes = 1	Staff and Guidelines Readiness Score = $\frac{T1 + T2}{2} \times 100$

	T2	Availability of PEN guidelines (observed)	No = 0<br>Yes = 1	
Equipment	T3	Availability of functioning BP set (observed)	No = 0<br>Yes = 1	Equipment Readiness Score $= \frac{T3 + T4 + T5}{3} \times 100$
	T4	Availability of weight measuring equipment (observed)	No = 0<br>Yes = 1	
	T5	Availability of height measuring equipment (observed)	No = 0<br>Yes = 1	
Essential Medicines	T6	Availability of metformin	No = 0<br>Yes = 1	Essential Medicines Readiness Score = $\frac{T6 + T7 + T8}{3} \times 100$<br><br>Essential Medicines Readiness (for peripheral facilities) Score = $\frac{T6 + T8}{2} \times 100$
	T7	Availability of glimepiride (Not Applicable for peripheral health facilities)	No = 0<br>Yes = 1	
	T8	Availability of dextrose	No = 0<br>Yes = 1	
Diagnostic Services	T9	Blood Glucose	No = 0<br>Yes = 1	Diagnostic Services Readiness Score = $\frac{T9 + T10 + T11}{3} \times 100$<br><br>Diagnostic Services Readiness (for peripheral facilities) Score = T9 × 100
	T10	Urine Protein (Not Applicable for peripheral health facilities)	No = 0<br>Yes = 1	
	T11	Urine Ketone (Not Applicable for peripheral health facilities)	No = 0<br>Yes = 1	
Diabetes Service Readiness =				

$$\frac{Staff\ and\ Guidelines + Equipment + Essential\ Medicines\ Diagnostic\ Services\ Rea\text{[illegible]}}{4}$$

Table S3: Number of participants by methods

**Participant group**	**Data collection method**	**Number**	**Number of participants**
Female elderly group (60 years and above)	FGDs	1	11
Female adult group (35-60 years)	FGDs	2	24
Male adult group (60 years and above)	FGDs	2	10
Female community health volunteers	FGDs	1	9
NCD patients female	IDIs	5	5
NCD patients male	IDIs	4	4
Community gatekeepers	Social mapping	2	43
Community gatekeepers	Transect walk	2	5
City officials and health providers	KII	15	15
Total			126

Table S4: Characteristics of individual in-depth interview and focus group discussions

**Characteristics**	**Categories**	**FGD**	**IDI**
Gender	Male	9	4
	Female	36	5
Age	20-39 years	14	1
	40-59 years	18	1
	60 and above	13	7
Education	No formal education	10	4
	Can read and write	15	5
	Primary Education	14	-
	Secondary Education	6	-
Occupation	Home makers	25	1

	Agriculture	8	1
	Business	4	1
	Labor	8	2
	Not engaged	-	4

Table S5: Characteristics of key informants interview (KII) participants

**Characteristics**		**n**
Gender	Male	10
	Female	5
Age	20-39 years	6
	40-59 years	9
	Pokhara health system actors	5
Work type	Health care providers	10
Work experience	<5 years	6
	> 5 years	9

Table S6: Public and private health facilities in urban and peri-urban areas of PMC.

**Health Provider Type**	**Category[1]**	**peri urban, n = 163, n (%)**	**urban, n = 497, n (%)**	**Overall, n = 660, n (%)**
**Public**		**36 (22.1%)**	**17 (3.4%)**	**53 (8.0%)**
Public Hospital	Allopathic care	3 (1.8)	1 (0.2)	4 (0.7)
Primary Health Care Centre	Allopathic care	2 (1.2)	0 (0.0)	2 (0.3)
Health Post	Allopathic care	18 (11.0)	1 (0.2)	19 (2.9)
Urban Health Clinic	Allopathic care	10 (6.1)	13 (2.6)	23 (3.5)
Urban Promotion Centre	Allopathic care	0 (0.0)	1 (0.2)	1 (0.2)
TB centre	Specialized care	0 (0.0)	1 (0.2)	1 (0.2)

[1] Categories include:

- Allopathic care: Modern Western medicine using drugs, surgery and diagnostics.
- Non-allopathic care: Traditional/alternative systems (e.g., Ayurveda, Homeopathy).
- Specialized care: Focused services for specific conditions or diagnostics by trained experts.

Ayurvedic Dispensary	Non-allopathic care	2 (1.2)	0 (0.0)	2 (0.3)
Public Ayurvedic Hospital	Non-allopathic care	1 (0.6)	0 (0.0)	1 (0.2)
**Private**		**127 (77.9%)**	**480 (96.6%)**	**607 (92.0%)**
Hospital/Nursing Home	Allopathic care	1 (0.6)	27 (5.4)	28 (4.2)
Clinic	Allopathic care	12 (7.4)	30 (6.0)	42 (6.4)
Polyclinic	Allopathic care	8 (4.9)	19 (3.8)	27 (4.1)
Pharmacy	Allopathic care	89 (54.6)	265 (53.3)	354 (53.6)
Ayurvedic Hospital	Non-allopathic care	0 (0.0)	6 (1.2)	6 (0.9)
NGO facility	Specialized care	1 (0.6)	4 (0.8)	5 (0.8)
Lab and Diagnostic Centre	Specialized care	0 (0.0)	21 (4.2)	21 (3.2)
Dental hospital/clinic	Specialized care	11 (6.7)	66 (13.3)	77 (11.7)
Ayurvedic Pharmacy	Non-allopathic care	5 (3.1)	30 (6.0)	35 (5.3)
Homeopathic Pharmacy	Non-allopathic care	0 (0.0)	8 (1.6)	8 (1.2)
Physiotherapy	Specialized care	0 (0.0)	1 (0.2)	1 (0.2)
Fertility Centre	Specialized care	0 (0.0)	3 (0.6)	3 (0.5)[2]

Table S7: Characteristics of health facilities providing diabetes and hypertension services in PMC (n=134)

**Health Facility Type**	**n (%)**
**Private = 85 (63.4%)**	
Private Hospital/ Nursing Home	26 (19.4%)
Private clinics	59 (44.0%)
**Public = 49 (36.6%)**	
Public Hospital	4 (3.0%)
Public primary healthcare facilities (PHC centres, urban health clinics and health promotion centres, health posts)	45 (33.6%)
Total facilities providing hypertension and diabetes services in PMC: Per population (PMC population: 513,540)	134<br>1 facility per 3832 population

Table S8: Joint display table of Hypertension and diabetes among the urban poor

Theme	Mapping	Social mapping/ transect walk in informal settlements/slum housing	Interviews/FGDs with urban residents in informal settlements/slum housing	Interviews with health providers/city authorities in Pokhara Metropolitan City	Health Facility Assessments	Key messages
**Knowledge, screening and diagnosis**	N/R	N/R	Diabetes and hypertension seen as a natural part of aging. At least one case, in every household. This led to a belief that the problems are common and should not be worried much.<br><br>Not wanting to be diagnosed through fear of addiction and long-term costs of taking medication.<br>Lack of trust in Dr's level of knowledge, inability to respond to patients' questions<br><br>Participants reported various health problems in their community that included heart related disease, high blood pressure, diarrheal disease, respiratory disease, diabetes, uterine prolapse etc. Terminologies: diabetes as Chinirog or Sugar while hypertension is referred to Pressure or high BP showing their understanding focused on causes and ways of management but hardly one could mention signs and symptoms and any complications if remain untreated.	N/R	Diabetes: 64% of public primary care services assessing blood glucose; 4.4% staff trained and having guidelines (4.4%).<br>92% of private primary care able to assess blood glucose, 3.4% staff training and none had guidelines available<br>Hypertension: all public and private had BP gauge, although limited staff training and guideline availability. Hospitals better equipped.	While facilities, public and private (more) have diagnostic equipment, few are trained. Communities are aware of this and lack trust in Drs. This, coupled with fears of taking costly medication and a belief that diabetes and hypertension are a natural part of aging and very common, reduces the chances that they will go to an allopathic provider for screening and diagnosis. Qualitative findings confirmed quantitative findings and provided and expanded understanding of the implications of limited training among health professionals for screening and diagnosis.
**Management of hypertension and diabetes**	Consistency of care – public facilities have more consistent management	Participants pointed out traditional healers and pharmacies and reported the practice within the community of visiting traditional healers when	Few had knowledge on when to go for screening or the complications and long-term health implications of either condition.<br>Differing opinions on need to take medication regularly. Younger females	Health providers reported lack of training, and high turnover of staff, so any trained staff are moved on.	All (49) public and 88% of private facilities (excluding specialist and non-allopathic) i.e 134 in total were providing HT/D2	Despite all public and a high proportion of private facilities providing HT/D2 services, there was a low preparedness score, challenges in the regular supply of medication

	scores, private was more variable.	their health problem was not considered serious. For conditions that were more urgent or concerning, they visited health facilities.	more likely to talk about taking hypertension medication regularly. Participants' adherence to the prescription and advice of health service providers varied. They expressed different ways of either acting on or resisting doctors' advice and treatment. Those who resisted were sceptical if the medicine would work at all and bound by addictive practices such as tobacco use. Some older participants felt medication should cure them, and reluctant to take medicines long-term. Common to turn to herbal or ayurvedic medicine when western medicine doesn't provide a cure.	Community health workers/volunteers had a good understanding of patients' perceptions and confirmed that some see symptoms of diabetes and HT as 'evil eye' when they are not cured immediately.<br><br>Patients likely to wait for symptoms to become serious and then go to hospital, by-passing primary care.<br><br>A few health workers commented on differences with rural settings where relatives and neighbours would ensure patients accessed treatment and support regularly.<br><br>Unanimously, all health care providers in public health facilities admitted they do not offer services as per the PEN package mainly due to lack of trained staff, guideline, equipment, and commodities. Importantly, delivering health services was seen as more challenging in urban areas due to inadequate services and	diagnosis and management services 49% of the 354. pharmacies were providing HT D2 services<br>Low overall preparedness to manage hypertension (overall median score of 44.4, IQR 44.4 to 55.6) and diabetes (62.5, IQR 50.0 to 75.0). Lowest scores for training and guidelines. Highest for equipment (also diagnosis above) limited availability of essential medications for hypertension at both public and private primary care clinics<br>Hospitals better prepared.	and limited trained staff to manage hypertension and diabetes on an on-going basis. As a result, patients were more likely to visit pharmacies for medication, if medication seen not to 'work' they traditional healers used. Clinics and hospitals only used when symptoms become serious. Ongoing management to prevent complications and worsening of symptoms within primary care was unlikely and possibly more challenging than in rural areas.<br><br>Findings came from all data sources. Qualitative findings confirm that many are not clear on how to take medication, or willing to take it long-term reflecting HFA findings showing lack of medication, training and ability of health professionals to provide appropriate support and advice, and stock-outs at both public and primary facilities.

				training and lack of community networks		
**Preventive behaviour**	N/R	Limited availability of fresh fruit and veg. Many small bars used by male daily wage labourers after work for drinking/smoking	Due to distrust of medicines, some relying on diet and yoga, but not checking BP or glucose.<br>Good knowledge of risk factors of poor diet, physical inactivity, and smoking.<br>Men and women report men to have high alcohol and smoking in response to heavy work, stress and low incomes.<br>Chewing tobacco common for women and seen as stress relief<br>Use of Yoga but partly driven by lack of belief in medicines.	Health workers reported not being trained to provide any counselling or support for people to quit smoking, alcohol or change diets.	Lack of guidelines and training available	While many understood the need to stop smoking, reduce alcohol and improve diets, attempts to change individual practices were undermined by the urban environment and working patterns. Health providers lack the skills and training to advise and support patients to adopt healthier lifestyles.<br>Qualitative findings support HFA findings and qualitative interviews with health professionals on lack of effective counselling on healthy behaviours.<br>Data from all sources, apart from mapping
**Access to care**	Private facilities and public hospitals are located in the centre, but public facilities in peri urban. Denser pop in centre, so public facilities less accessible.	Participatory ranking: Pharmacy use is highest due to ease of access, availability of medicines, low cost and more guidance given. Similar scores for traditional healers, public health facilities and private health facilities, with variations depending on seriousness. Hospitals and private clinics sought if very serious.	Pharmacies seen as friendly and easy to use, compared to long distances and waiting times to get to public primary care and hospitals. Daily-wage workers can only use pharmacies as need to take time out of work to access public primary care opening hours. Pharmacies start early and open late.<br>Out-of-pocket payments in pharmacy or private clinics as health insurance premiums seen as too costly.	City decision-makers (PMC) and health facility managers agreed that the number and spread of public primary care was insufficient to meet the needs of the growing urban population and that public services were more limited than in rural areas.<br><br>Health insurance only used by middle-class, although there have been some schemes to	Only 8% of facilities were public. Majority of private facilities (53%) are pharmacies. followed by dental clinics (11.7%), private clinics (6.4%), private hospitals (4.2%) and private polyclinics (4.1%).<br>21 types of health provider<br>Plus 173 pharmacies	Public health facilities are few and mainly located in peri-urban areas. Despite attempts to roll-out health insurance, low-income urban residents are most likely to visit pharmacies, unless their condition is serious, due to the ease of access in terms of proximity and opening hours which fit with long hours of daily-wage workers.<br>Data from all sources.<br>Confirmation of high cost of

				offer free registration as a political motivation.<br><br>Health workers in the community are aware that pharmacies offer convenient service hours compared to public health facilities that makes it more accessible to urban poor.		insurance premiums confirmed by professionals and residents. All sources confirm high use of pharmacies and private facilities. Qualitative and mapping highlight both proximity and cost and opening hours.
**Population and planning for rising NCDs**	No measure of deprivation at small area, the maps can only show population density and need to be guided by estimates by PMC staff of where slums are.	Complexity of defining and identifying urban poor. Intersections of age/occupation/location are important. Occupation is key to reflect their economic status like daily wage labour in construction sites, working as porters. For some, people living in poor housing settlements like slum areas were the identifier, but many others disagreed as individuals with white-collar jobs also reside in slum areas, and urban poor distribution is distributed beyond the slum areas	Intersections e.g. with age and disability. Poverty of individuals within households particularly those with disability or elderly people relying on others or governmental allowance for their survival. Communities referred to them as the ultra-poor.	Across all the interviews with the health system actors and health care providers, they accepted the fact that local health system does not have any formal mechanism to identify and reach out to urban poor community. Despite budget allocation is made for poor and marginalized section of the community, it is usually the recommendation of the locally elected representatives to recommend an individual as poor to get benefit from the offered services, however, its mostly judgmental and lacks any defined mechanism.<br><br>PMC officials identified slum areas within the city	N/R	There is no means to target services to poor households or areas within the city. While city officials can identify slum areas, communities in those areas emphasised the lack of homogeneity with ultra-poor unable to support themselves as well as white-collar workers within slum areas.<br><br>Data from all sources apart from HRA. Contradiction between qualitative data from city officials and participatory and resident interviews on the homogeneity of slums.

Text S1. Explanation of authorship change statement

During the manuscript submission process, the designations of co-first authors were not clearly indicated. As the final confirmation of the authors' respective contributions which determined the co-first authorship was conducted subsequent to the submission procedure, this designation could not be incorporated at the time of upload. The authors regret this omission and any inconvenience it may have caused.

The author HE made substantial contributions to this work – conceptualised the study, co-wrote the first draft of the manuscript, contributed in developing the study methodology and tools, contributed in performing qualitative data analysis, validated and supervised the study, and approved the final version. We acknowledge this lack of clarity as our responsibility and sincerely apologise for the confusion. All authors have reviewed and agreed to this correction, have provided dated manuscript versions showing the authors' contributions, and we confirm that the requested changes accurately reflect each author's contributions in accordance with the Journal's authorship policies. We appreciate the Journal's understanding and consideration of this request.
